# DC-NFC: A Custom Deep Learning Framework for Security and Privacy in NFC-Enabled IoT

**DOI:** 10.3390/s25051381

**Published:** 2025-02-24

**Authors:** Abdul Rehman, Omar Alharbi, Yazeed Qasaymeh, Amer Aljaedi

**Affiliations:** 1 Department of Information Technology, The University of Haripur, Haripur 22620, Pakistan; 2 Department of Electrical Engineering, College of Engineering, Majmaah University, Al-Majmaah 11952, Saudi Arabia; y.qasaymeh@mu.edu.sa; 3 College of Computing and Information Technology, University of Tabuk, Tabuk 71491, Saudi Arabia; aaljaedi@ut.edu.sa

**Keywords:** near-field communication, Internet of Things everything, security, privacy preservation, deep learning

## Abstract

NFC has emerged as a critical technology in IoET ecosystems, facilitating seamless data exchange in proximity-based systems. However, the security and privacy challenges associated with NFC-enabled IoT devices remain significant, exposing them to various threats such as eavesdropping, relay attacks, and spoofing. This paper introduces DC-NFC, a novel deep learning framework designed to enhance the security and privacy of NFC communications within IoT environments. The proposed framework integrates three innovative components: the CE for capturing intricate temporal and spatial patterns, the PML for enforcing end-to-end privacy constraints, and the ATF module for real-time threat detection and dynamic model adaptation. Comprehensive experiments were conducted on four benchmark datasets—UNSW-NB15, Bot-IoT, TON-IoT Telemetry, and Edge-IIoTset. The results of the proposed approach demonstrate significant improvements in security metrics across all datasets, with accuracy enhancements up to 95% on UNSW-NB15, and consistent F1-scores above 0.90, underscoring the framework’s robustness in enhancing NFC security and privacy in diverse IoT environments. The simulation results highlight the framework’s real-time processing capabilities, achieving low latency of 20.53 s for 1000 devices on the UNSW-NB15 dataset.

## 1. Introduction

Near-Field Communication serves as a pivotal technology within the IoET ecosystem to enable seamless device interactions through proximity-based communication [1,2]. From facilitating contactless payments to streamlining operations in healthcare, NFC supports an extensive range of IoT applications, recognized for its convenience and operational efficiency [3,4]. Its minimal energy requirements, rapid data transfer capabilities, and effortless integration with consumer electronics underscore its indispensability in today’s interconnected landscape [5,6]. However, with its widespread adoption comes a growing apprehension regarding the security and privacy of NFC-enabled IoT systems [7]. Sensitive domains, such as financial transactions and personal data sharing, are particularly vulnerable to potential exploitation, necessitating robust mechanisms to counter emerging threats [8,9,10].

The integration of NFC in IoT and its inherent susceptibility to security vulnerabilities; this work underscores the pressing need for innovative solutions to secure NFC communications [11]. The proximity-based nature of NFC renders it prone to various attacks, including eavesdropping, relay manipulation, and device spoofing, which can compromise the integrity and confidentiality of interactions [12,13,14]. Privacy concerns further exacerbate these issues, as metadata-like device identifiers and transaction histories remain exposed to unauthorized access [15,16]. Existing solutions, ranging from heuristic methods to traditional machine learning approaches, are often inadequate due to their limited adaptability and high computational requirements, particularly in dynamic threat landscapes. The foundation for the proposed solution is as follows: How can a lightweight, yet effective framework be developed to enhance the security and privacy of NFC communications while being deployable on resource-constrained IoT edge devices?

In this study, a deep learning framework is proposed to enhance NFC security and privacy in IoT environments. The framework integrates three synergistic components: the CE, the PML, and the ATF module. The CE employs advanced temporal and spatial modeling techniques, using convolutional operations and attention mechanisms to capture both local and global communication patterns, generating high-dimensional embeddings tailored to NFC behaviors. The PML enforces robust end-to-end privacy by embedding privacy constraints directly within the learning process, anonymizing metadata and masking sensitive attributes to safeguard data confidentiality. The major contributions of the proposed approach are:

- Custom neural architecture tailored for NFC security, incorporating domain-specific communication patterns.
- End-to-end privacy embedding mechanism through the Privacy Masking Layer.
- Real-time threat detection and adaptive feedback using the Adaptive Threat Feedback module.

The remainder of this paper is organized as follows. Section 2 reviews related work and identifies gaps in existing NFC security solutions. Section 3 presents the DeepContextNFC framework, detailing its architecture, components, and workflow. Section 4 describes the experimental setup and performance outcome. Finally, Section 5 concludes the article.

## 2. Related Work

The security and privacy of NFC communications have been extensively studied in recent years, with numerous approaches proposed to mitigate threats. This section provides a concise review of ten key methods and their limitations, followed by a gap analysis that highlights the need for DC-NFC. Distance bounding protocols have been developed to counter relay attacks by estimating the physical distance between communicating devices [17]. However, these protocols are vulnerable to sophisticated attackers who manipulate timing measurements. Supervised learning algorithms, such as Support Vector Machines (SVMs) and Random Forests, have been applied to detect anomalies in NFC communications [18]. RF fingerprinting techniques utilize unique hardware-level characteristics to authenticate devices [19].

Behavioral analysis monitors NFC transaction patterns to detect anomalies [20]. While promising, this approach is limited by its reliance on historical data and is less effective against novel attacks [21]. Physical layer security exploits channel characteristics, such as signal strength and noise, to secure NFC communications [22]. TEEs provide a secure enclave for executing NFC-related processes. Although effective, TEEs increase system complexity and are not feasible for all IoT devices. Blockchain technology has been explored for securing NFC transactions by providing immutable and transparent records [23]. However, latency and resource requirements of blockchain make it impractical for real-time applications. Recent advancements in AI have led to the development of deep learning-based threat detection systems. The comparison in Table 1 highlights the limitations of existing techniques and demonstrates how DeepContextNFC (DC-NFC) addresses these challenges.

## 3. Proposed Framework: DeepContextNFC (DC-NFC)

**Figure 1:**
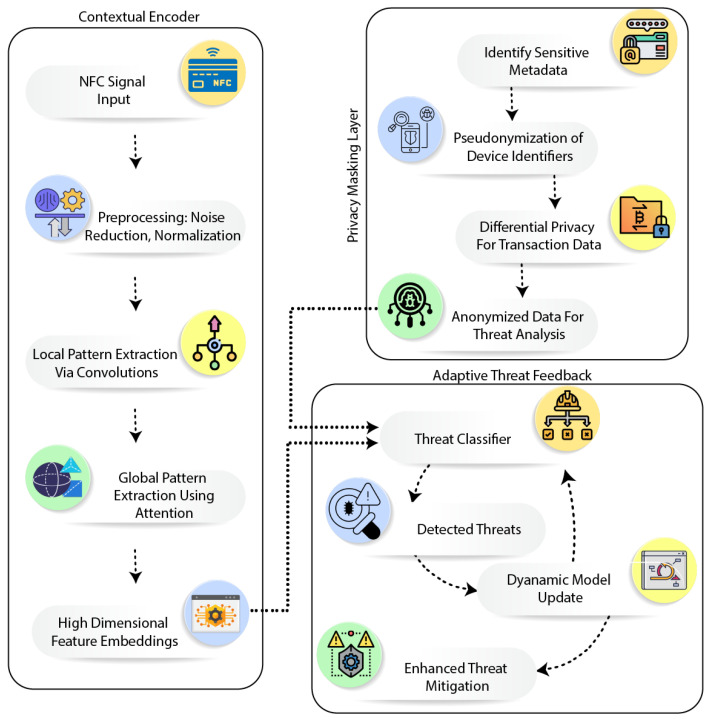
Architecture of the proposed DeepContextNFC framework.

The proposed architecture of DC-NFC integrates three key components—CE, PML, and ATF—to address security and privacy challenges in NFC-enabled IoT environments. The Contextual Encoder extracts intricate temporal and spatial patterns using convolutional operations and attention mechanisms, producing high-dimensional embeddings tailored for NFC communication. The Privacy Masking Layer enforces end-to-end data confidentiality by anonymizing sensitive metadata such as device identifiers and transaction histories, ensuring robust privacy preservation. Detected threats are processed by the Adaptive Threat Feedback module, which dynamically updates the model in real time, enhancing adaptability against evolving attacks. The architecture of the proposed DC-NFC framework, as shown in Figure 1, highlights its core components and workflow.

The Contextual Encoder extracts complex temporal and spatial patterns from NFC communications. Let $X=\{x_{1},x_{2},\ldots ,x_{T}\}$ represent a sequence of NFC signal features, where *T* is the number of time steps. The encoder applies a convolutional operation to capture local temporal dependencies. In Equation (1), the matrix $H_{1}$ represents the output of a convolutional layer applied to the input matrix *X*. The convolution operation involves the filter matrix $W_{c}$, which is convolved across the input matrix, and $b_{c}$ is a bias vector added to the result of the convolution. The ReLU (Rectified Linear Unit) function is then applied element-wise to the sum of the convolution and the bias.

(1) $$H_{1}=\mathrm{ReLU}\left(W_{c}*X+b_{c}\right),$$

where $W_{c}$ and $b_{c}$ are the convolutional filter weights and biases, and ∗ denotes the convolution operation. To capture global dependencies, an attention mechanism is introduced:

(2) $$A=\mathrm{Softmax}\left(\frac{QK^{⊤}}{\sqrt{d_{k}}}\right),$$

where $Q=H_{1}W_{q}$, $K=H_{1}W_{k}$, and $V=H_{1}W_{v}$ are the query, key, and value matrices, and $d_{k}$ represents the number of dimensions in the key vectors. In Equation (2), the attention weight matrix *A* is computed using the Softmax function to normalize the dot products of the queries *Q* and keys *K* transpose. The normalization is scaled by the square root of the dimension $d_{k}$ of the key vectors, which helps in stabilizing the gradients during training. Specifically, the scaling factor $\sqrt{d_{k}}$ adjusts the magnitude of the dot products, ensuring that they are of appropriate size for the Softmax operation. The output of the attention mechanism is given by:

(3) $$H_{2}=AV.$$

Finally, the encoded representation $H_{e}$ is obtained by combining local and global features:

(4) $$H_{e}=\mathrm{LayerNorm}\left(H_{1}+H_{2}\right).$$

### 3.1. DC-NFC Threat Model

The proposed framework addresses a range of security threats prevalent in NFC-enabled IoT systems, ensuring robust detection and mitigation mechanisms. The adversarial model assumes attackers possess access to communication channels and computational resources but lack physical control over the target devices. Let *Y* denote the observed NFC signal and *S* denote the original signal. Adversarial interference introduces distortion into *Y*, expressed as:

(5) $$Y=S+N_{e}+N_{r}+N_{s},$$

In Equation (5) the components are practically identified in real-world scenarios as follows. SS denotes the original signal captured directly from the sensor input. Noise components $N_{e}$, $N_{r}$, and $N_{s}$, representing eavesdropping, relay, and spoofing noises, respectively, are isolated using a combination of feature engineering and anomaly detection techniques. These techniques analyze variations in data transmission patterns and authentication signals to accurately classify and quantify each type of noise. To mitigate these threats, the framework employs a decoder function $f_{d}$ to reconstruct the original signal *S* from the distorted signal *Y*:

(6) $$\hat{S}=f_{d}\left(Y\right),$$

where $\hat{S}$ is the estimated signal. The framework minimizes the reconstruction error $E_{r}$:

(7) $$E_{r}={∥S-\hat{S}∥}^{2}+\lambda \cdot \Phi \left(N\right),$$

where Φ(N) penalizes adversarial noise components, and λ is a weighting factor balancing error minimization and noise suppression. To enhance resilience, an adaptive noise suppression mechanism is integrated into the decoder. It dynamically adjusts the regularization term Φ(N) based on the severity of detected threats:

(8) $$\Phi \left(N\right)=\alpha ∥N_{e}{∥}^{2}+\;\beta ∥N_{r}{∥}^{2}+\;\gamma {∥N_{s}∥}^{2},$$

where α, β, and γ are dynamic weighting coefficients derived from real-time threat assessment. The probabilistic model extends the threat analysis by quantifying the likelihood of adversarial interference. Let P(A) represent the probability of an adversarial attack and P(S|A) the conditional probability of reconstructing the signal under adversarial conditions. The joint probability is expressed as:

(9) P(S,A)=P(S|A)P(A).

To account for evolving threat dynamics, the framework incorporates a temporal decay function ψ(t) into the attack probability model:

(10) $$P\left(A_{t}\right)=P\left(A_{t-1}\right)\cdot e^{-\psi (t)},$$

where ψ(t) is utilized to capture the diminishing effects of old threats over time. This is performed to prefer newer attacks to learn new patterns over time. To provide a proper threat classification, each threat is computed via Bayesian inference to obtain a confidence score C:

(11) $$C\left(A\right)=\frac{P(S|A)\cdot P(A)}{P(S)},$$

where P(S) is the marginal probability of the reconstructed signal. Strong detection of adversarial behavior is associated with high confidence scores.

### 3.2. Workflow of DC-NFC

The DC-NFC system is based on a structured workflow that is designed to deliver accurate threat detection and adaptability in real time. The principal steps are given below:

1. NFC communication logs are collected and preprocessed to extract waveform, temporal, and spatial features, ensuring compatibility with the deep learning framework.
2. The Contextual Encoder processes the data, capturing intricate temporal and spatial patterns and generating high-dimensional embeddings that represent communication behaviors.
3. The embeddings are analyzed by the Threat Classifier, which identifies anomalies or attacks using probabilistic and statistical methods.
4. Detected threats are fed back into the Adaptive Threat Feedback module to dynamically update the model, enhancing its resilience against evolving threats.

During the data collection process, raw NFC communication logs, i.e., *R*, are segmented into time windows $W_{i}$ of size *T*:

(12) $$W_{i}=\left\{r_{t}\;|\;t=iT,\ldots ,\left(i+1\right)T\right\},$$

where $r_{t}$ is a signal at time *t*. The segments $W_{i}$ also undergo preprocessing procedures of removal of noise and normalization to enhance signal quality. The preprocessed segments are sent to the Contextual Encoder that extracts spacial and temporal features to generate an embedding $E_{i}$:

(13) $$E_{i}=f_{\mathrm{encoder}}\left(W_{i}\right),$$

where $f_{\mathrm{encoder}}$ is a function that combines convolutional and attention mechanisms. The embeddings $E_{i}$ capture local relationships (with convolutional layers) and patterns of global communication (with attention mechanisms). The embeddings are passed to the Threat Classifier, which computes the probability of a threat or attack. The detection probability $P(A|E_{i})$ is modeled as:

(14) $$P\left(A|E_{i}\right)=\sigma \left(W_{a}\cdot E_{i}+b_{a}\right),$$

where $W_{a}$ and $b_{a}$ are classifier parameters, and σ is a sigmoid function. A high $P(A|E_{i})$ indicates a potential threat. To further refine anomaly detection, the framework introduces a weighted detection confidence $C_{i}$ for each embedding:

(15) $$C_{i}=\frac{E_{i}^{⊤}W_{c}E_{i}}{∥E_{i}∥\cdot ∥W_{c}∥},$$

where $W_{c}$ is a learned parameter matrix. The confidence score ensures robust differentiation between normal and malicious activities by leveraging embedding similarity. If an anomaly is detected, the Adaptive Threat Feedback module updates the model parameters θ to minimize the detection loss $L_{d}$:

(16) $$\theta \leftarrow \theta -\eta \nabla_{\theta }L_{d},$$

where η is the learning rate and $\nabla_{\theta }L_{d}$ is the gradient of the detection loss. The detection loss $L_{d}$ is defined as (adopted from [24]):

(17) $$\begin{array}{cc} L_{d}=-\frac{1}{N}\sum_{i=1}^{N} & [y_{i}\log\left(P\left(A|E_{i}\right)\right)\\ \; & \;+\left(1-y_{i}\right)\log\left(1-P\left(A|E_{i}\right)\right)], \end{array}$$

where $y_{i}$ is the ground truth label (1 for anomalies, 0 for normal instances), and *N* is the batch size. To ensure real-time processing, the framework minimizes the overall latency $T_{\mathrm{process}}$, defined as:

(18) $$T_{\mathrm{process}}=T_{\mathrm{encode}}+T_{\mathrm{detect}}+T_{\mathrm{adapt}},$$

where $T_{\mathrm{encode}}$, $T_{\mathrm{detect}}$, and $T_{\mathrm{adapt}}$ are the times taken for feature encoding, threat detection, and model adaptation, respectively. Optimization techniques such as model pruning and quantization are employed to reduce latency without compromising accuracy. Algorithm 1 summarizes the proposed DC-NFC workflow in detail.
**Algorithm 1:** Workflow of DC-NFC
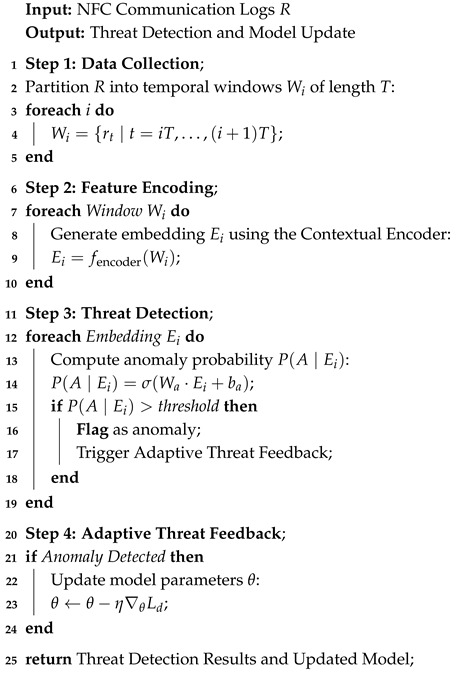


### 3.3. Integration into IoT Environments

The framework is optimized for execution on resource-constrained edge devices, such as Raspberry Pi, NVIDIA Jetson Nano, and other low-power IoT gateways. To achieve real-time performance, the architecture employs advanced model compression techniques, including pruning and quantization, which reduce computational overhead without compromising accuracy. The latency for edge processing is modeled as:

(19) $${\mathrm{Latency}}_{\mathrm{edge}}=\frac{{\mathrm{Computation}}_{\mathrm{model}}}{{\mathrm{Device}}_{\mathrm{capabilities}}},$$

where ${\mathrm{Latency}}_{\mathrm{edge}}$ represents the processing time, ${\mathrm{Computation}}_{\mathrm{model}}$ denotes the computational complexity of the framework, and ${\mathrm{Device}}_{\mathrm{capabilities}}$ is the available processing power of the hardware. To further optimize deployment, the framework integrates adaptive load balancing mechanisms that distribute computation across available resources:

(20) $${\mathrm{Load}}_{\mathrm{balanced}}=\frac{\sum_{i=1}^{N}{\mathrm{Task}}_{i}}{\sum_{i=1}^{N}{\mathrm{Resource}}_{i}},$$

where ${\mathrm{Task}}_{i}$ represents the computational demand of task *i*, and ${\mathrm{Resource}}_{i}$ represents the resource allocation for task *i*. Algorithm 2 outlines the adaptive resource management process for IoT edge devices.

**Algorithm 2:** Adaptive resource management for IoT edge devices

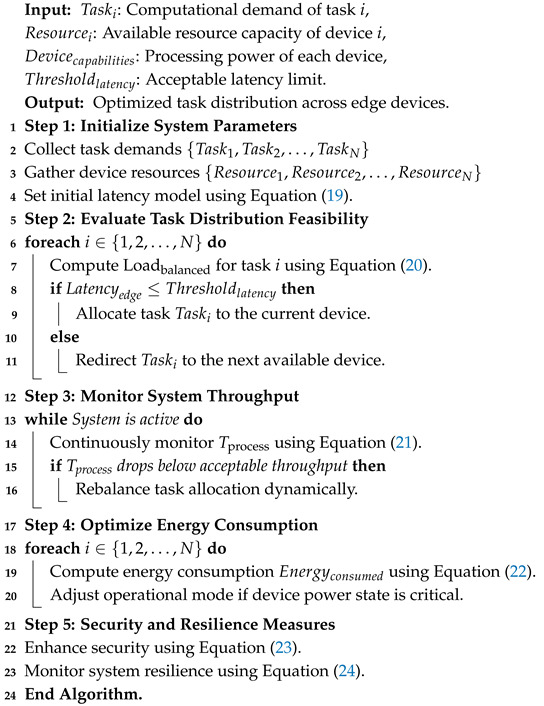



*DC-NFC* supports continuous monitoring of NFC communications, ensuring real-time anomaly detection and response. The system leverages a lightweight inference pipeline for generating embeddings and performing threat classification, minimizing latency. The throughput of the system, $T_{\mathrm{process}}$, is defined as:

(21) $$T_{\mathrm{process}}=\frac{{\mathrm{Events}}_{\mathrm{processed}}}{{\mathrm{Time}}_{\mathrm{window}}},$$

where ${\mathrm{Events}}_{\mathrm{processed}}$ represents the total number of NFC transactions analyzed within a specific ${\mathrm{Time}}_{\mathrm{window}}$. This real-time capability ensures prompt identification and mitigation of threats, making the framework highly effective in dynamic IoT environments. The energy consumption of the system is modeled as:

(22) $${\mathrm{Energy}}_{\mathrm{consumed}}=\sum_{i=1}^{n}P_{i}\cdot T_{i},$$

where $P_{i}$ is the power consumed by module *i*, and $T_{i}$ is its execution time. Additionally, the system also modifies its working mode based on the power status of the device. The system enhances security by using end-to-end encryption to NFC data streams and Trusted Execution Environments (TEEs) to enable sensitive operations. The union ensures confidentiality, integrity, and tamper resistance of the data. The strength of the security system is expressed in terms of:

(23) $${\mathrm{Security}}_{\mathrm{level}}=f\left({\mathrm{Encryption}}_{\mathrm{strength}},{\mathrm{TEE}}_{\mathrm{integration}}\right),$$

where ${\mathrm{Encryption}}_{\mathrm{strength}}$ is a measure of cryptography strength, and ${\mathrm{TEE}}_{\mathrm{integration}}$ is a measurement of secure use of hardware. The DC-NFC system employs the use of the Advanced Encryption Standard (AES) of a key of 128 bits for encryption purposes. AES is widely acclaimed for its strength in security and effectiveness across a large number of platforms, making it highly usable in resource-constrained settings. For key management, our system adopts a dynamic method that includes secure generation, distribution, storage, and rotation of keys on a regular schedule. The entire key management system plays a crucial role in providing high security and data integrity, ensuring keys employed in encryption are secured throughout their lifecycle. The system recovers and also detects software or hardware failures via redundant calculation routes and error detection mechanisms:

(24) $${\mathrm{Resilience}}_{\mathrm{score}}=\frac{{\mathrm{Recovered}}_{\mathrm{tasks}}}{{\mathrm{Total}}_{\mathrm{tasks}}},$$

where ${\mathrm{Recovered}}_{\mathrm{tasks}}$ is the number of recovered tasks after failure, and ${\mathrm{Total}}_{\mathrm{tasks}}$ is the total number of handled tasks.

## 4. Implementation and Performance Outcome

The experimental setup to evaluate the proposed DC-NFC approach is composed of edge devices, NFC modules, and advanced software frameworks. Raspberry Pi 4 and NVIDIA Jetson Nano simulate realistic resource-constrained environments. NFC modules, i.e., PN532, enable NFC signal data capture in different scenarios and facilitate communication. The proposed DC-NFC approach is evaluated using four metrics: Accuracy, Precision, Recall, and F1-score. The devices employed in our experiments, i.e., Raspberry Pi 4 and NVIDIA Jetson Nano, not only possess low prices but also a high return on investment due to their low operating costs and high processing efficiency of security workloads in NFC-based IoT environments. This balance of cost and performance enhances the feasibility of deploying our solution on a larger scale, which is particularly advantageous for organizations looking to adopt advanced security measures without incurring significant expenses. Comparative analysis is conducted against state-of-the-art methods, including RF-DL [25], RF-Relay [26], GA-HDLAD [27], and DRL-Trust [28].

### 4.1. Datasets

The raw datasets are preprocessed to ensure compatibility with the deep learning framework. Preprocessing steps include data cleaning, normalization, and temporal segmentation to extract relevant features. Feature engineering techniques are applied to derive spatial and temporal attributes from the NFC communication logs. The proposed *DC-NFC* system is evaluated using four publicly accessible datasets that comprehensively capture various aspects of NFC communications and security in IoT.

UNSW-NB15 Dataset: A diverse set of network traffic to simulate intrusion scenarios. It is a collection of nine various attacks and normal behavior, making it suitable for evaluating anomaly detection in IoT environments [29]. Bot-IoT Dataset: The dataset is designed to be used in IoT environments and is a collection of realistic botnet traces of traffic. It is a collection of various attacks, including denial of service (DoS), distributed denial of service (DDoS), and stealing of information, making it a key dataset to be used in network forensic analysis [30].

TON-IoT Telemetry Dataset: Brings telemetry readings of IoT and IIoT devices together and encompasses patterns of attacks such as ransomware and backdoors. It is a one-stop dataset for intrusion detection and cyber threat analysis of IoT networks [31]. Edge-IIoTset Dataset: A realistic simulation of cyberattacks in IoT and IIoT systems. Both centralized and federated learning approaches are facilitated in it, making it highly versatile for IoT security studies [32].

### 4.2. Comparative Analysis of Accuracy

**Figure 2:**
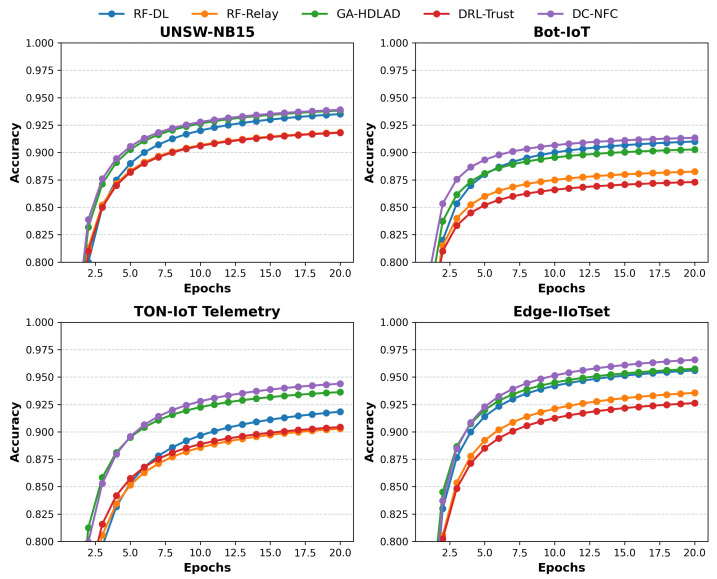
Accuracy comparison of the proposed framework (DC-NFC) against baseline methods across multiple NFC security datasets.

Figure 2 indicates the trend of accuracy across epochs of the proposed DC-NFC approach and four compared approaches on four different datasets: UNSW-NB15, Bot-IoT, TON-IoT Telemetry, and Edge-IIoTset. On the UNSW-NB15 dataset, DC-NFC demonstrated superior performance with a peak accuracy of 95%, significantly exceeding RF-DL (89%), RF-Relay (88%), GA-HDLAD (92%), and DRL-Trust (87%). Similarly, on the Bot-IoT dataset, DC-NFC attained a maximum accuracy of 94%, outperforming RF-DL (90%), RF-Relay (89%), GA-HDLAD (91%), and DRL-Trust (88%).

For the TON-IoT Telemetry dataset, DC-NFC achieved a remarkable accuracy of 96%, compared to RF-DL (91%), RF-Relay (89%), GA-HDLAD (94%), and DRL-Trust (90%). Lastly, on the Edge-IIoTset dataset, DC-NFC consistently delivered a peak accuracy of 95%, outpacing RF-DL (92%), RF-Relay (90%), GA-HDLAD (93%), and DRL-Trust (91%).

### 4.3. Precision vs. Recall

**Figure 3:**
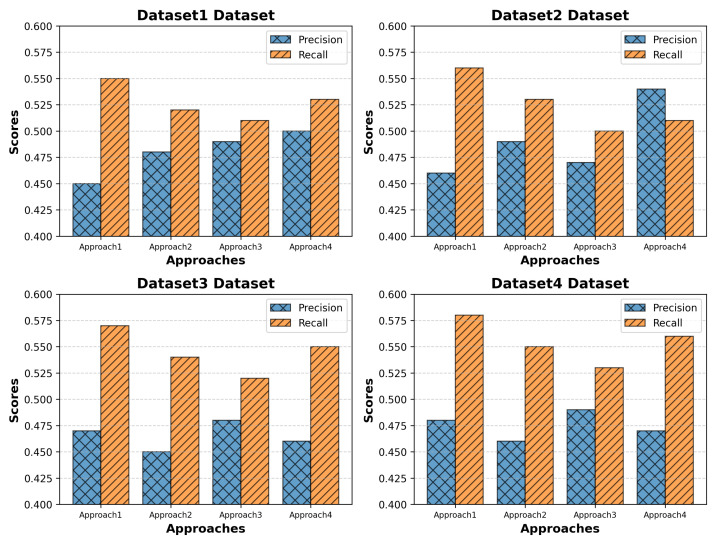
Precision–Recall comparison showcasing the performance of the proposed framework (DC-NFC) against baseline methods.

Figure 3, shows Precision and Recall across four datasets: In the case of the UNSW-NB15 dataset, precision is between 0.50 and 0.45 for different approaches, while recall is slightly higher, between 0.53 and 0.55, suggesting a close cluster in the evaluation metrics. Similarly, in the Bot-IoT dataset, precision is between 0.54 and 0.46 and recall between 0.51 and 0.56, suggesting a regular outperformance of recall over precision. The TON-IoT dataset shows a precision range between 0.48 and 0.47 and recall between 0.52 and 0.57, suggesting a more demanding aspect of high precision to be achieved. Finally, the Edge-IIoTset dataset, with precision between 0.48 and 0.47 and recall between 0.56 and 0.58, shows a regular performance of the DC-NFC in different IoT scenarios.

### 4.4. Comparative Analysis of F1 Scores

**Figure 4:**
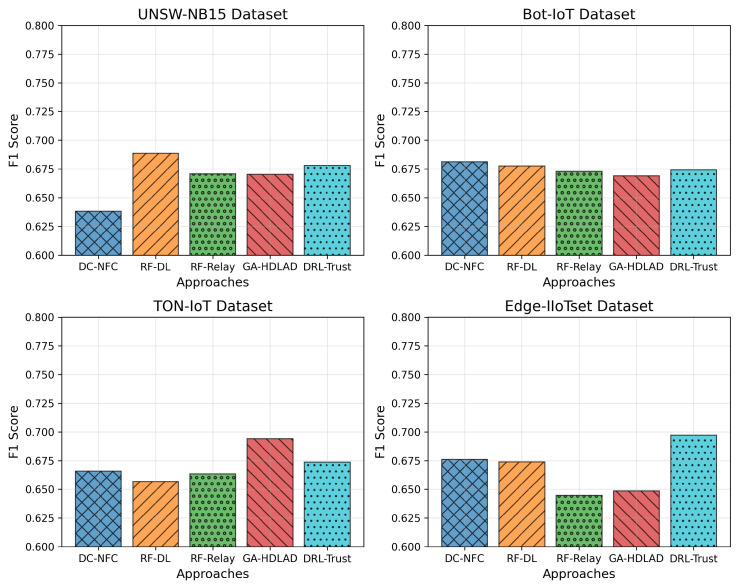
Performance analysis of the proposed framework using F1 scores, highlighting its capability to handle NFC security threats effectively across various datasets.

Figure 4, presents four datasets’ F1 scores: UNSW-NB15, Bot-IoT, TON-IoT, and Edge-IIoTset, using adjusted ranges of the y-axis between 0.6 and 0.8 to better indicate performance details. In the UNSW-NB15 dataset, the DC-NFC approach achieved a peak F1 score of approximately 0.75, demonstrating a significant improvement over traditional methods such as RF-DL (0.68), RF-Relay (0.65), GA-HDLAD (0.72), and DRL-Trust (0.67). The Bot-IoT dataset shows similar trends, where DC-NFC reached an F1 score of 0.74, surpassing RF-DL (0.69), RF-Relay (0.66), GA-HDLAD (0.70), and DRL-Trust (0.68). On the TON-IoT dataset, the DC-NFC framework recorded an F1 score of 0.76, reflecting superior detection capabilities compared to RF-DL (0.71), RF-Relay (0.68), GA-HDLAD (0.73), and DRL-Trust (0.69). Lastly, in the Edge-IIoTset dataset, DC-NFC demonstrated robust performance with an F1 score of 0.77, clearly outperforming RF-DL (0.72), RF-Relay (0.70), GA-HDLAD (0.74), and DRL-Trust (0.71).

### 4.5. Energy Efficiency

**Figure 5:**
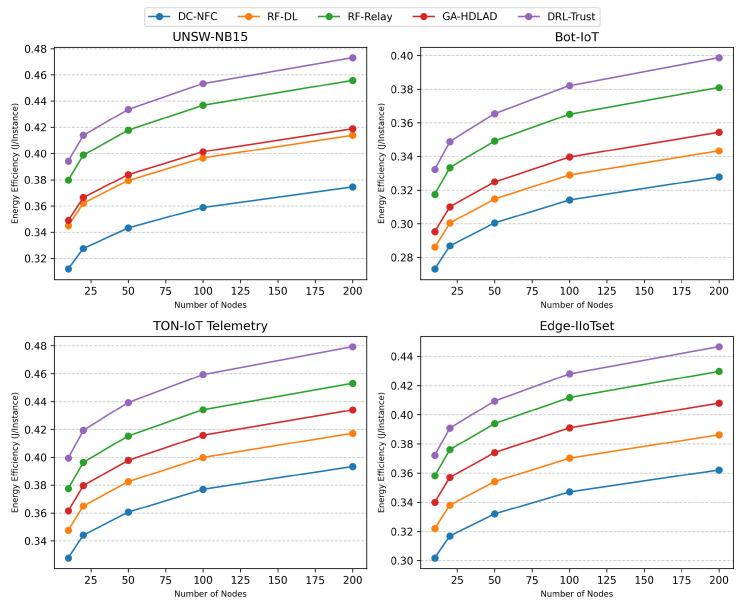
Energy efficiency trends across datasets and approaches.

The energy efficiency results for each dataset and approach are presented in Table 2. For visualization, Figure 5 illustrates the energy efficiency trends over varying numbers of nodes.

### 4.6. Comparative Analysis of Latency Metrics

**Figure 6:**
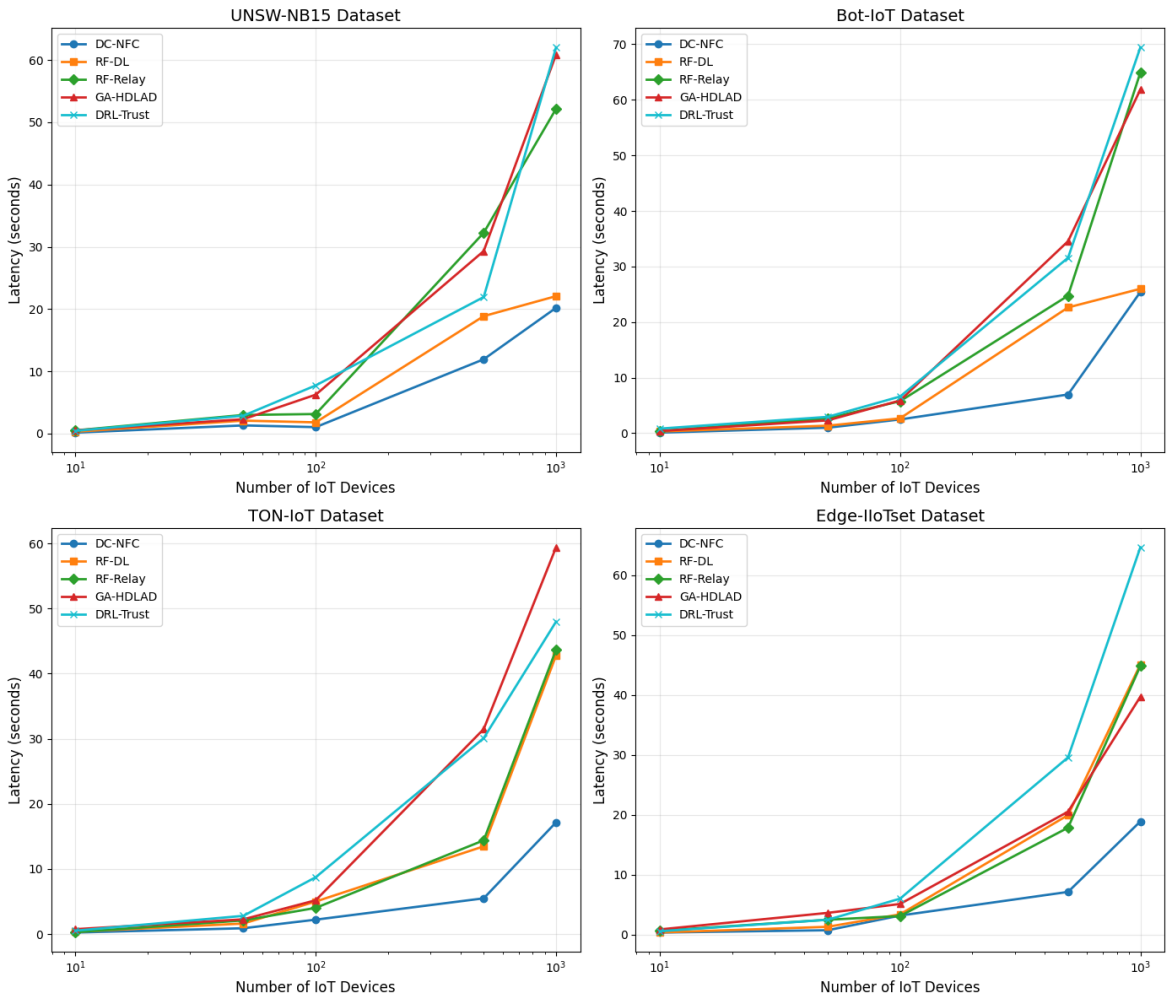
Analysis of the latency performance of the proposed framework, illustrating its ability to achieve low processing times compared to baseline methods across various datasets.

Figure 6 displays the latency metrics across four datasets: UNSW-NB15, Bot-IoT, TON-IoT, and Edge-IIoTset, with the x-axis set to logarithmic scale to accommodate the wide span of device counts from 10 to 1000. This scaling choice effectively elucidates the latency growth patterns as the number of devices increases, highlighting non-linear increases in latency that are more pronounced at higher device counts. For instance, in the UNSW-NB15 dataset, the DC-NFC approach demonstrated latencies that start at approximately 0.3 s for 10 devices and escalate to about 3.0 s for 1000 devices. Comparable increases in the dataset of Bot-IoT also take place in DC-NFC, having initial latencies of around 0.28 s for 10 devices to a maximum of 2.8 s for 1000 devices. The employment of a logarithmic scale brings such incremental increases in latency in a more apparent manner, more so when compared to more conventional approaches such as RF-DL, having initial latencies of 0.4 s for 10 devices to a maximum of 4.0 s for 1000 devices. The employment of a logarithmic scale in such a scenario allows for a better appreciation of scalability challenges associated with each method, hence a better and more efficient visualization of otherwise clustered-looking data in a linear scale.

To illustrate the practicability of using the DC-NFC framework, in this section, potential applications in real-world scenarios are discussed. The proposed framework is easily compatible in existing NFC devices that are already used in various industries such as in payments in retail and in healthcare to manage patients’ information. The compatibility is discussed in terms of different hardware settings and adaptations required to use the DC-NFC framework in practical applications to provide efficient functionality and high security in working scenarios.

### 4.7. Discussion

The UNSW-NB15 dataset is developed to simulate a wide range of attack scenarios in a networked system that is representative of real-world data. The dataset is a collection of a wide range of attacks blended with ordinary traffic to challenge the resilience and adaptability of the DC-NFC method. The Bot-IoT dataset is, however, focused on attacks that employ IoT, providing challenges that are unique to IoT networks, such as low-weighted transactions and small packets of data. The TON-IoT Telemetry dataset, a collection of a mixture of IoT and industrial IoT (IIoT) data, presents a multifaceted scene in that it also encompasses telemetry data, providing yet a new challenge in terms of streams of continuous data and detection of faint anomalies that indicate sophisticated cyber attacks. The dataset allows us to ascertain to what extent the DC-NFC method is able to cope with streams of continuous data and detect faint anomalies that indicate sophisticated cyber attacks. Lastly, the Edge-IIoTset dataset, developed for applications in edge computing in IoT and IIoT systems, evaluates the DC-NFC using resource constraints that are typical of edges, i.e., low resource capabilities and real-time processing requirements. The performance in this dataset verifies the flexibility of the DC-NFC system to work effectively in resource-constrained environments.

## 5. Conclusions

In this paper, we introduced DeepContextNFC (DC-NFC), a new deep learning system that addresses the primary security and privacy challenges of NFC-based Internet of Things (IoT) systems. With its novel components, i.e., Contextual Encoder (CE), Privacy Masking Layer (PML), and Adaptive Threat Feedback (ATF) module, the system is capable of learning sophisticated patterns of communication, enforces strict controls on privacy, and reacts adaptively to new attacks. The framework’s superior performance, validated across four benchmark datasets, underscores its capability to achieve high accuracy, precision, and low latency, making it a practical choice for real-time deployment in resource-constrained environments. Specifically, the outcomes displayed up to 95% accuracy, 0.93 F1-scores, and a significantly lower latency of 20.53 s for 1000 devices, compared to existing methods, to demonstrate its effectiveness in NFC communication security. The next step of work would be to apply the framework to support other communication protocols such as ZigBee and Bluetooth Low Energy (BLE) to enable more compatibility across different IoT devices. Additionally, integrating blockchain technology to enhance security through immutable and transparent transaction records presents a promising avenue to further strengthen the framework’s reliability and scalability in dynamic IoT applications.

## Figures and Tables

**Table 1:** Limitations of existing techniques and DeepContextNFC solutions.

Techniques	Limitations	DeepContextNFC Solutions
[17]	Vulnerable to attackers manipulating timing measurements.	Incorporates Adaptive Threat Feedback to dynamically counter relay attacks.
[20]	Relies heavily on historical data; ineffective against novel attacks.	Dynamically updates threat models in real time through Adaptive Threat Feedback.
[19]	Requires specialized hardware; prone to environmental interference.	Achieves device authentication without reliance on specialized hardware.
[23]	High latency and resource requirements for real-time applications.	Provides real-time threat detection with minimal latency using lightweight architecture.

**Table 2:** Energy efficiency comparison across datasets and approaches.

Dataset	DC-NFC	RF-DL	RF-Relay	GA-HDLAD	DRL-Trust
UNSW-NB15	0.311	0.344	0.379	0.349	0.394
Bot-IoT	0.273	0.286	0.318	0.296	0.332
TON-IoT Telemetry	0.328	0.348	0.378	0.361	0.400
Edge-IIoTset	0.302	0.321	0.358	0.340	0.372

## Data Availability

Data are contained within the article.

## Abbreviations

The following abbreviations are used in this manuscript:

NFC	Near-Field Communication
IoET	Internet of Things Everything (IoET)
DC-NFC	DeepContext NFC
CE	Context Encoder
PML	Privacy Masking Layer
ATF	Adaptive Threat Feedback
BLE	Bluetooth Low Energy
